# Reconciling Allergy Information in the Electronic Health Record After a Drug Challenge Using Natural Language Processing

**DOI:** 10.3389/falgy.2022.904923

**Published:** 2022-05-10

**Authors:** Ying-Chih Lo, Sheril Varghese, Suzanne Blackley, Diane L. Seger, Kimberly G. Blumenthal, Foster R. Goss, Li Zhou

**Affiliations:** ^1^Division of General Internal Medicine and Primary Care, Department of Medicine, Brigham and Women's Hospital, Boston, MA, United States; ^2^Harvard Medical School, Boston, MA, United States; ^3^Mass General Brigham, Boston, MA, United States; ^4^Division of Rheumatology, Allergy, and Immunology, Department of Medicine, Massachusetts General Hospital, Boston, MA, United States; ^5^Department of Emergency Medicine, University of Colorado School of Medicine, Aurora, CO, United States

**Keywords:** clinical decision support system (CDSS), electronic health record (EHR), drug challenge test, medication reconciliation, natural language processing

## Abstract

**Background:**

Drug challenge tests serve to evaluate whether a patient is allergic to a medication. However, the allergy list in the electronic health record (EHR) is not consistently updated to reflect the results of the challenge, affecting clinicians' prescription decisions and contributing to inaccurate allergy labels, inappropriate drug-allergy alerts, and potentially ineffective, more toxic, and/or costly care. In this study, we used natural language processing (NLP) to automatically detect discrepancies between the EHR allergy list and drug challenge test results and to inform the clinical recommendations provided in a real-time allergy reconciliation module.

**Methods:**

This study included patients who received drug challenge tests at the Mass General Brigham (MGB) Healthcare System between June 9, 2015 and January 5, 2022. At MGB, drug challenge tests are performed in allergy/immunology encounters with routine clinical documentation in notes and flowsheets. We developed a rule-based NLP tool to analyze and interpret the challenge test results. We compared these results against EHR allergy lists to detect potential discrepancies in allergy documentation and form a recommendation for reconciliation if a discrepancy was identified. To evaluate the capability of our tool in identifying discrepancies, we calculated the percentage of challenge test results that were not updated and the precision of the NLP algorithm for 200 randomly sampled encounters.

**Results:**

Among 200 samples from 5,312 drug challenge tests, 59% challenged penicillin reactivity and 99% were negative. 42.0%, 61.5%, and 76.0% of the results were confirmed by flowsheets, NLP, or both, respectively. The precision of the NLP algorithm was 96.1%. Seven percent of patient allergy lists were not updated based on drug challenge test results. Flowsheets alone were used to identify 2.0% of these discrepancies, and NLP alone detected 5.0% of these discrepancies. Because challenge test results can be recorded in both flowsheets and clinical notes, the combined use of NLP and flowsheets can reliably detect 5.5% of discrepancies.

**Conclusion:**

This NLP-based tool may be able to advance global delabeling efforts and the effectiveness of drug allergy assessments. In the real-time EHR environment, it can be used to examine patient allergy lists and identify drug allergy label discrepancies, mitigating patient risks.

## Introduction

Beta-lactam antibiotics are the most frequently cited causative agent for medication-related allergies, accounting for nearly 10–20% of drug hypersensitivity reactions recorded in electronic health records (EHRs) (1, 2); however, a vast majority of patients with a reported beta-lactam allergy do not have a true immunological reaction that warrants an allergy label (3–5). Furthermore, inaccurate allergy labels for antibiotics contribute to growing antimicrobial resistance (6). Therefore, there is a greater urgency to incorporate beta-lactam allergy evaluation in antimicrobial stewardship programs (7–10).

Due to the high prevalence of inappropriate allergy labels, drug challenge tests are an integral part of the drug allergy assessment. A drug challenge, also termed a “test dose,” “graded challenge,” or “drug provocation test” in allergy literature, is the administration of a drug under medical observation. Sousa-Pinto et al. demonstrated that penicillin allergy testing would significantly reduce costs for both inpatient and outpatient care (11). Similarly, testing other highly utilized drug classes that may cause hypersensitivity reactions, including analgesics and antimicrobials, will likely minimize avoidable adverse events and healthcare expenditures that result from alternative treatments (2).

Still, drug challenge tests are not successfully optimized in patient care due to logistical and clinical barriers. For one, there are limited specialists and materials to conduct testing (9). In addition to resource limitations, after a specialist performs a challenge test, the results are not automatically updated to the patient allergy list (12). Consequently, allergy specialists performing the tests must remember to enter the allergy module to update the results at the conclusion of the visit, which is not part of their usual clinical note documentation workflow. As a result, other clinicians may not have the most updated allergy information prior to prescribing medications. Even if the clinical note is read by a non-allergist, clinicians may not feel it's their responsibility to remove allergens from the allergy list despite their knowledge of negative challenge test results. In addition, Rimawi et al. also reported 36% of patients had penicillin allergy redocumentation some time later without obvious reason even though the patient has a prior negative drug challenge test result (13). Conversely, allergens may occasionally be removed despite a positive historical drug challenge test, which could pose a risk to patient safety. Thus, it is imperative to ensure that the detailed drug allergy information from challenge tests is considered in allergy reconciliation and maintaining an updated allergy list.

Given these challenges, we developed the Allergy Reconciliation Module, a clinical decision support system, that is part of a larger initiative to enhance allergy documentation in the EHR and reconcile discrepancies in the patient's allergy record.

## Methods

### Clinical Setting and Data Sources

Approved by the Mass General Brigham (MGB) Institutional Review Board, this study was conducted using EHR data from the Enterprise Data Warehouse (EDW) of MGB, a large non-profit hospital and physician network in the northeastern US. We specifically used historical structured and unstructured EHR data for patients who received a drug challenge test in outpatient settings between June 5, 2015 and July 31, 2020 to develop the challenge test interpretation rule for our NLP-based tool. For these patients, demographic information, allergy lists, flowsheets, and clinical notes were analyzed.

### Drug Challenge Test Interpretation

**Figure 1:**
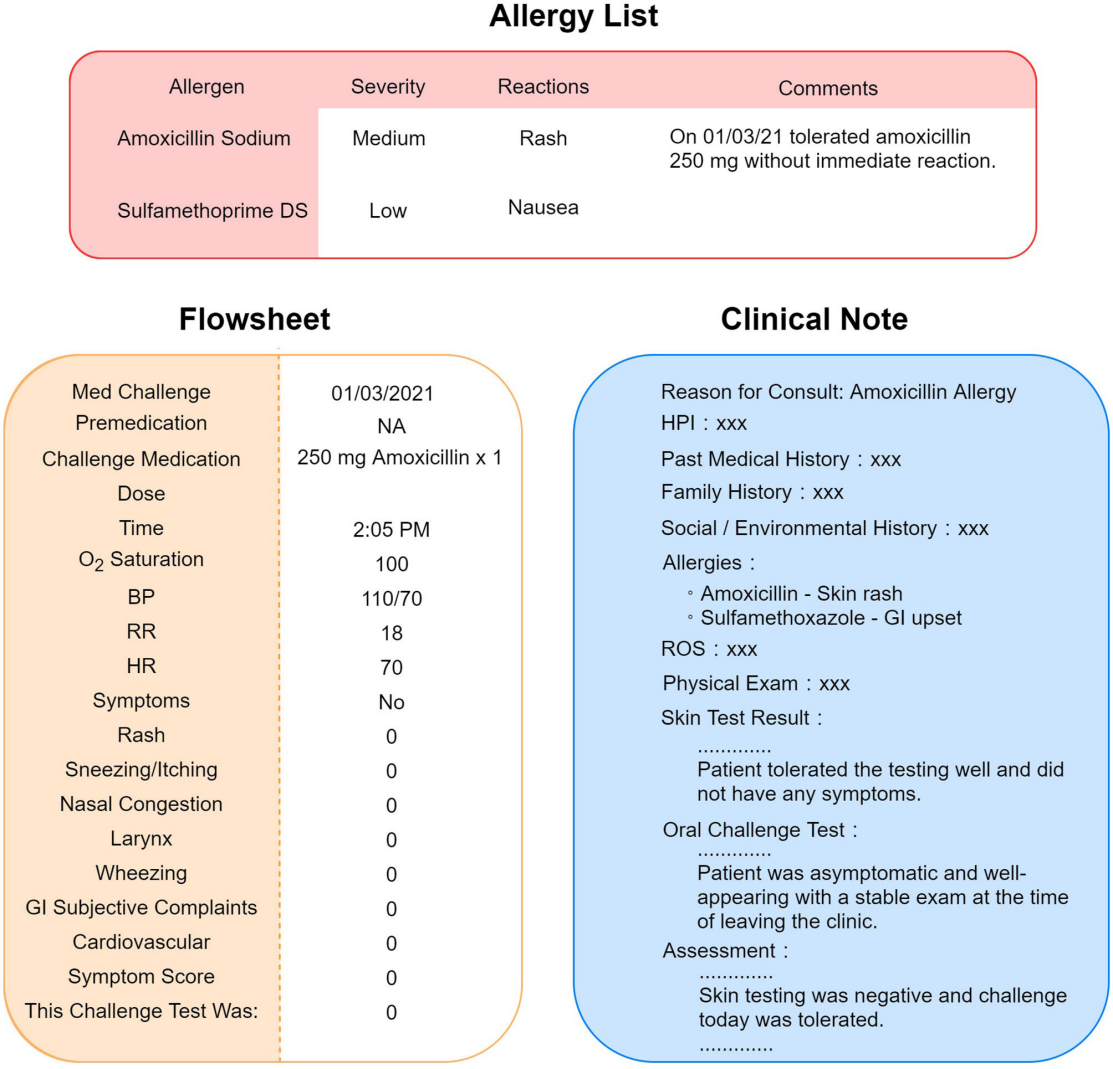
Scenario of allergy information discrepancy in EHR. Allergy list, flowsheets, and clinical notes are different locations in the EHR that store allergy information. A negative result of drug challenge test may not be updated to the allergy list accordingly. Sometimes, the physician would leave a comment instead of removing the allergen from the list, as pictured in this figure.

To interpret the results of drug challenge tests, we integrated two sources of information, flowsheets and clinical notes, to extract and process the drug challenge test results. Flowsheets are structured EHR data, which provide detailed information about the challenge test, including the date/time the challenge test was performed, premedications (if given), medication name, dosage, and result of the testing; if there is a reaction, then it is detailed with the type, severity, vital signs and physical examination findings. Because the drug name was recorded in a free text format, we processed the drug name to convert it into structured data to be consistent with other flowsheet information (Figure 1). Given that not all data fields in a flowsheet may be filled out, we developed an NLP algorithm to interpret the result of the challenge test based on the clinical notes authored by allergy specialists to uncover more discrepant allergy records.

**Figure 2:**
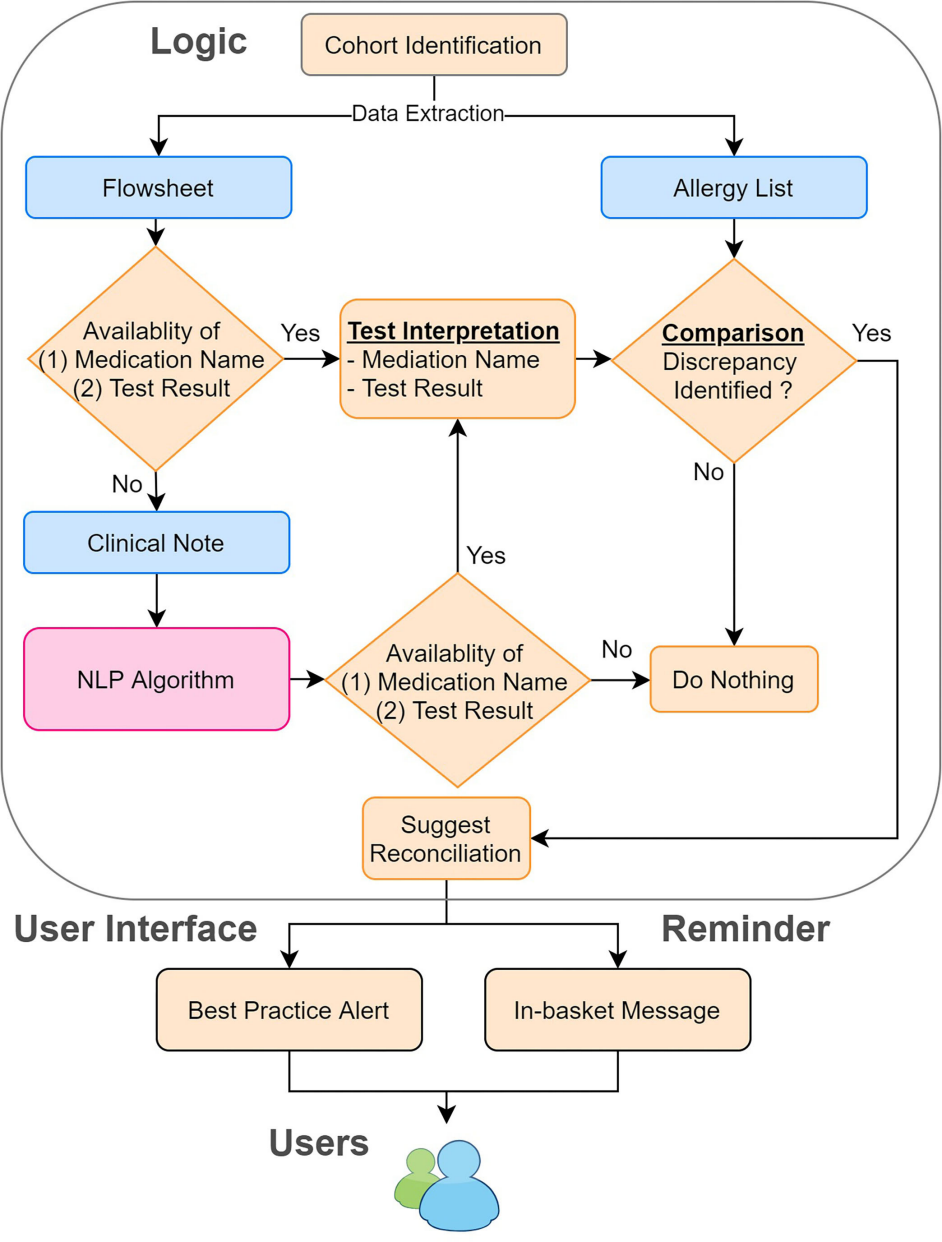
System architecture of the reconciliation module. We combined the information derived from the flowsheets and clinical notes. We then compared this information to the allergy list to identify the discrepancies. If any discrepancies were found, we sent in-basket messages weekly to remind the physician to reconcile the allergy discrepancies by using our tool.

To process clinical notes, we applied rule-based NLP to extract the name of the testing medication and the result of the drug challenge test. First, we retrieved clinical notes in the Hyper Text Markup Language (HTML) format from the production environment of EPIC (Verona, WI), the EHR system for MGB. To improve the efficiency and accuracy of our NLP program, we filtered out irrelevant clinical notes, such as notes related to food challenge tests, using keyword searches. Then, we applied our NLP ecosystem (MTERMS, Medical Text Extraction, Reasoning and Mapping System) (14) to extract the challenge agent and the result. To avoid inaccurate recommendations, we intentionally suppressed the NLP algorithm output when the note was highly challenging to interpret. For example, notes that are very lengthy with historical allergy information or contain information of prior challenge test present commonplace difficulties for NLP to process. Finally, we integrated the result derived from flowsheets and the NLP algorithm together with flowsheets as the prioritized information source and NLP alone as the backup information source. The overall system architecture is shown in Figure 2.

### Allergy List Discrepancy Identification

**Figure 3:**
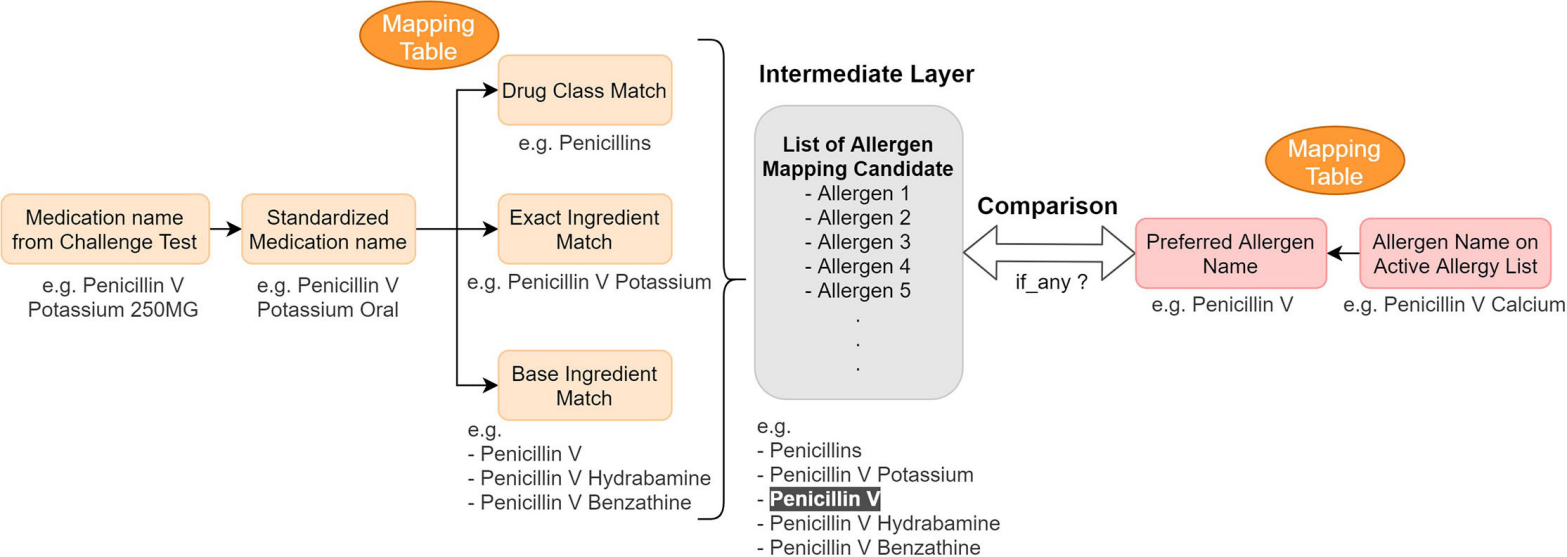
Concept mapping for the reconciliation mechanism—logic and example. We used mapping tables to map (1) medication name in a challenge test to different drug hierarchy relationship (2) active allergen to their preferred name. Then we compared them to find any information discrepancy.

After consolidating challenge test results from several data sources in the EHR, we developed mapping tables to identify discrepancies between the EHR allergy list and the processed challenge test results. The tables map the challenge agent to the allergen name on the patient's allergy list to determine whether any inconsistencies exist between the allergy list and the extracted challenge test results (Figure 3).

The algorithm's output and the mapping table together inform the final component of the reconciliation module—the clinical recommendations. If a discrepancy is identified based on the algorithm's output, the module is prompted to provide a reconciliation recommendation that highlights the discrepancy. We created another mapping table to map the medication name to the preferred allergen name so that the preferred name can be used in our recommendation module when we suggest adding a new allergen to the allergy list. If no discrepancies are identified, the module will not suggest any action. In order to avoid erroneous recommendations, we did not provide reconciliation recommendations for cases that were difficult to interpret due to ambiguous results (e.g., subjective symptoms only, non-allergic symptoms such as headache or dry mouth) or delayed reactions not observed during the clinical observation period.

### Evaluation of NLP Algorithm

To assess the performance of our NLP algorithm, we evaluated 200 randomly sampled specialists notes associated with drug challenge tests that were performed between June 5, 2015 and January 5, 2022. Notes with an extremely short length (character count < 1,000) were excluded from the evaluation corpus since such notes contained limited information for interpreting the test results. We manually reviewed the patients' allergy lists, flowsheets, and clinical notes to compare the interpretation result from each data source and generate a confusion matrix. The precision was calculated based on the confusion matrix. Finally, we calculated the rate of allergy discrepancies detected by our module (i.e., recall) and the actual rate of allergy discrepancies. Additionally, we calculated the availability of interpretable challenge test information from each data source due to the variable amount of information available in solely flowsheets or clinical notes. To estimate the impact of the tool on a larger note corpus, we used the tool to estimate the number of discrepancies among the whole population to approximate how many allergen records could potentially be corrected by using our reconciliation module.

### System Integration and Implementation in EHR

**Figure 4:**
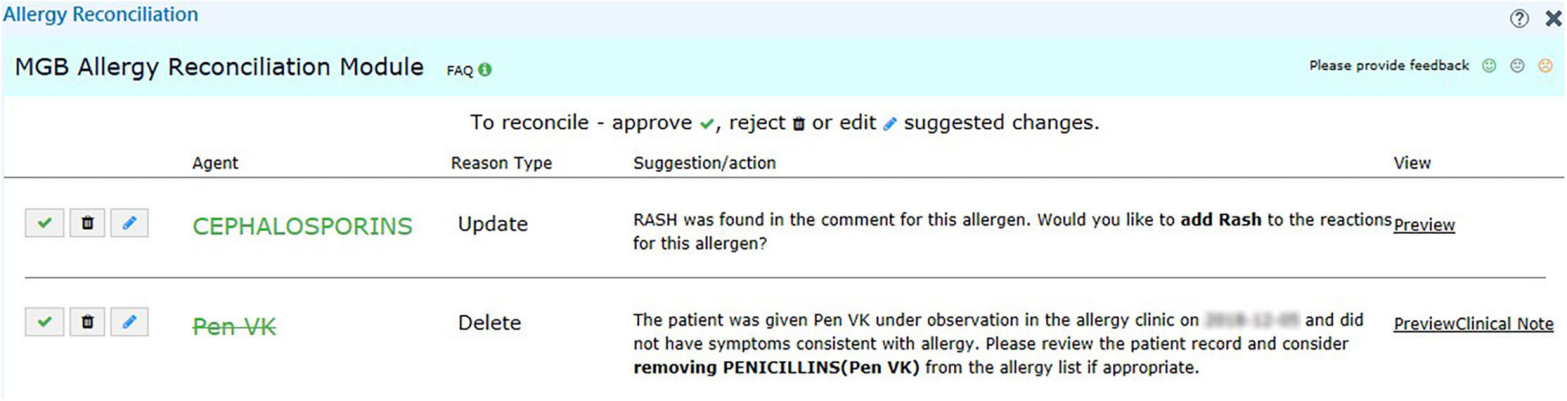
User interface of the recommendation for challenge tests. We provide the reason in addition to the suggested action, such as “Add” and “Delete” for the user to make decision. We also include a hyperlink (right-hand side) to the clinical notes in case the user wants to know more about the reaction.

We set up the testing, staging, and production environment for different development purposes in EPIC. In the testing environment, we validated the rule logic and evaluated the user workflow with mock data from testing patients. In the staging environment, we used actual patient data in the EPIC support (SUP) environment to further test our program. Any changes in the EPIC SUP environment would be retracted the next day and would not affect the data in the production environment, which clinicians engaged with regularly. The production environment was reserved for clinicians in our pilot group to obtain user feedback about the performance and usability of our module. To promote the usage of our tools, we also generated a report of discrepancy statistics and sent in-basket messages to physicians in EPIC to bring awareness to existing discrepancies on a weekly basis (Figure 2). The user interface for our recommendations is shown in Figure 4.

### Statistical Analysis

The means and standard deviations were reported for all continuous variables, including age and the number of drug allergy labels per patient. Independent *t*-tests were used to compare the age and number of drug allergy labels between the sampled and population cohorts. Fisher's exact test and Chi-square tests were used to compare the distribution of categorical variables, such as sex, race, and ethnicity. Statistical analyses were performed using R software (version 4.0.5). A *p* < 0.05 was considered statistically significant.

## Results

We identified 4,313 patients who had 5,312 notes and received drug challenge tests between June 9, 2015 and January 5, 2022 at MGB. The demographic characteristics of the patient cohort are shown in Table 1. The distribution of sex, race, ethnicity, and number of drug allergy labels per patient for the sampled and total population were similar although the sampled cohort was younger.

**Table 1:** Demographic characteristics of patients cohort received drug challenge test.

**Characteristics**	**Reviewed cohort**	**Total cohort**	***p* value^b^**
No of Notes	200	5,312	
No of Patients	197	4,313	
Age	48.9 ± 18.3	51.7 ± 18.8	0.03
Female	151 (76.6)	3,239 (75.1)	0.674
**Race** ^a^			0.713
White	169 (85.8)	3,649 (84.6)	
Black	4 (2.0)	143 (3.3)	
Asian	10 (5.1)	185 (4.3)	
Other/unknown	14 (7.1)	336 (7.8)	
Ethnicity, Hispanic^a^	8 (4.1)	219 (5.1)	0.618
No of Drug Allergy Labels per Patient	5.75 ± 6.11	5.82 ± 6.35	0.962

*Values are expressed as mean ± SD or number of patients (percent) based on patient level*.

a *Self-reported*;

b *Independent t test, Fisher's exact test or Chi-square test*.

To test the NLP algorithm, 200 encounter notes from 197 unique patients were randomly selected. Of these 200 notes concerning drug challenge tests, antibiotics were the primary challenge class, comprising 85% of all challenge agents (*n* = 170) and with penicillin being the most common (*n* = 117, 59%) (Table 2). Non-steroidal anti-inflammatory drugs (NSAIDs) were the second most frequently (6.5%) tested medication.

**Table 2:** Descriptive statistics of reviewed challenge test by drug class.

**Drug Class**	**No of challenge test**	**No of discrepancy**
	***n* = 200**	***n* = 14**
Antibiotics	170 (85.0)	10 (71.4%)
- Penicillin	118 (59.0)	5 (35.7%)
- Cephalosporin	16 (8.0)	0
- Sulfonamide	18 (9.0)	3 (21.4%)
- Quinolone	6 (3.0)	0
- Macrolide	6 (3.0)	0
- Tetracycline	2 (1.0)	0
- Gentamycin	1 (0.5)	0
- Vancomycin	1 (0.5)	1 (7.1%)
- Other antibiotics	2 (1.0)	1 (7.1%)
NSAIDs^#^	13 (6.5)	2 (14.3%)
Acetaminophen	1 (0.5)	1 (7.1%)
Prednisolone	3 (1.5)	0
Vaccine	2 (1.0)	0
Others^*^	11 (5.5)	1 (7.1%)

*Values are expressed as count (%)*.

# *NSAIDs, Non-steroidal anti-inflammatory drugs*.

* *Includes Metformin, Prednisolone, Tamoxifen, Lidocaine, Potassium, Tropicamide eye drop, Famciclovir, Progesterone, Plaquenil, Ondansetron, Methotrexate and Metoclopramide*.

We generated a confusion matrix (Table 3) and calculated the precision, recall (percentage of extractable challenge test information available) and percentage of discrepancies identified for the following sources: [1] Flowsheets, [2] NLP algorithm on clinical notes, and [3] Flowsheets and NLP Algorithm on clinical notes together. The precision was 100% for flowsheets, 96.1% for the NLP algorithm and 98.7% for the flowsheet + NLP algorithm. Forty-two percent, 61.5%, and 76.0% of the challenge test results could be determined using flowsheets alone, the NLP algorithm alone, or flowsheets and NLP algorithm together, respectively for these notes. Among the 200 randomly selected cases, 14 (7.0%) of the patient allergy lists were not updated to reflect the challenge test results. Flowsheets were used to identify 2.0% of these discrepancies, and the NLP algorithm alone detected 5.0% of these discrepancies from clinical notes. Due to the overlapping information in flowsheets and clinical notes, referring to flowsheets and NLP-extracted results together detected 5.5% of discrepancies.

**Table 3:** Confusion matrix of different allergy information sources.

**Ground truth**	**Allergy list**		**Flowsheet**			**NLP**^a^, ^b^			**Flowsheet** **+** **NLP**^a^, ^c^		
*n* = 200 notes	+	-	+	-	Undetermined	+	-	Undetermined	+	-	Undetermined
Positive (*n* = 2)	1	1	0	0	2	1	0	1	1	0	1
Negative (*n* = 198)	13	185	0	84	114	0	122	76	0	151	46

a *NLP, Natural Language Processing; There are also 5^b^ and 2^c^ false positive output due to capture the result of historical challenge test erroneously (not shown due to table's format)*.

To evaluate the impact and generalizability of our methods, we applied our algorithm to all clinical notes in the population (*n* = 5,312 notes). Flowsheet and NLP extracted information from the clinical notes identified 153 (2.9%) and 202 (3.8%) potential allergy discrepancies, respectively. Most of the potential discrepancies (>90%) were “Delete” recommendations, in which the allergy reconciliation module suggested removing the allergen (Table 4). While we did not perform manual chart review to validate all recommendations, the estimated number of discrepancies is expected to approximate the actual number of discrepancies since we have a high precision (flowsheet: 100%, NLP: 96.1%).

**Table 4:** Number of allergy discrepancies for each recommendation type.

**Recommendation**	**Reviewed notes** ^c^		**Total notes** ^b^	
	**(*****n*** **=** **200)**		**(*****n*** **=** **5,312)**	
**Count (%)**	**NLP** ^a^	**Flowsheet**	**NLP** ^**a**^	**Flowsheet**
Add	1 (0.5%)	0 (0%)	13 (0.2%)	8 (0.2%)
Delete	9 (4.5%)	4 (2%)	189 (3.6%)	145 (2.7%)
Total	10 (5%)	4 (2%)	202 (3.8%)	153 (2.9%)

a *NLP, Natural Language Processing*.

b *Estimated Number of Discrepancies*.

c *Exclude Notes with Short Length (No of Characters < 1,000)*.

Through error analysis, we identified scenarios that the NLP algorithm cannot process reliably. First, we found 5 patients who had a long history of prior allergy information in their clinical notes, which were unexpectedly captured by our tool and affected NLP-based results. Applying NLP logic alone in these cases resulted in a false positive rate of 2.5%. Notably, the false positive rate decreased to 1% when using information obtained from flowsheets and NLP results together, as indicated in Table 3. Second, an output containing a long list of challenge agents indicated that the patient had a complicated challenge test history, or the single note involved several challenge agents. Third, one patient developed a delayed reaction after the clinic visit, and another patient developed allergy symptoms again after re-taking the medication despite having a negative challenge test. In both cases, the NLP algorithm was not able to account for these delayed reactions. Lastly, we realized the clinical notes associated with the challenge test may not contain all the information our algorithm requires to produce interpretable results.

We subsequently modified the algorithm in several ways to address these complex patient scenarios and ensure the accuracy of the allergy reconciliation recommendations. We added post-processing logic to suppress recommendations when the algorithm identified more than two challenge agents. Recommendations to delete allergens are also suppressed if the module identifies any delayed allergy reactions mentioned in the free text comment field, which is often used by clinicians when documenting allergy information.

During the pilot testing of our tool, we provided clinicians access to the reconciliation module. The participating physicians saw an orange “MGB Allergy Reconciliation” button on the left-hand side of their screen when allergy discrepancies were found that required action by the clinician. Upon clicking the button, the users would see the recommended actions (such as “Add” and “Delete”) (Figure 4). If the user elected to understand the rationale behind the recommendation or action, a hyperlink would allow the user to pull up the associated clinical note side by side with the allergy reconciliation module. Because the user could submit feedback within the module, audited real-time user feedback and generated user statistics were captured from the backend. As our study is ongoing, we are still collecting usage and feedback data, which are not ready for analysis at this point.

## Discussion

In this study, we demonstrated the utility of an NLP-based tool that ensures allergies are properly recorded in the EHR and aligned with the results of drug challenge tests performed by specialists. By analyzing a subset of the patient population at MGB, nearly 7% of patients did not have an up-to-date, accurate EHR allergy list which poses a risk to patient safety. This allergy discrepancy rate is likely lower than the frequency of discrepancies found in other healthcare systems since MGB has a robust allergy program in place with more than 40 specialists who frequently provide allergy consultations and testing (15); MGB has performed more than 5,000 challenge tests that have been overseen by 47 specialists. Parkland Health and Hospital System in Texas, another similarly large healthcare institution, only conducted 17 penicillin tests in a 20-month span and identified 12.9% allergies that required relabeling prior to implementing allergy reconciliation interventions (16). Additionally, nearly a quarter of patients have an unknown or unverified documented penicillin allergy in EHRs, suggesting that the reported rate of discrepancies in allergy labels is likely an underestimate due to infrequent testing and reconciliation efforts (17, 18). Moreover, when a negative drug challenge test is not properly communicated, patients may be precluded from receiving clinically appropriate and more preferable treatments.

Drug challenge tests were most frequently performed to evaluate antibiotic allergies, largely, penicillin antibiotics. Since over half of drug challenge tests are dedicated to assessing penicillin allergies, these tests emphasize the growing concerted effort to delabel penicillin allergies. As discussed by Blumenthal et al., most patients labeled with penicillin allergy were not truly allergic to penicillin and passed the challenge test (19). We also found a positive result for a challenge test to be quite rare (~1%), confirming patients most likely do not have a true immunological reaction. The penicillin allergy may still be recorded because physicians do not actively remove the allergy or hesitate to delete allergens from the allergy list despite the negative challenge test result, propagating or allowing these discrepancies to persist. Even if the penicillins allergy has been delabelled, they may still be added back due to erroneous reentries (20). As one third of discrepancies are pencillin tests that are not appropriately communicated in the EHR allergy list, these discrepancies detract from delabeling efforts and antibiotic stewardship. Our multimodal approach directly applies to ensuring erroneous penicillin allergies are delabelled promptly using novel methods (8, 9, 16).

When reviewing the allergy discrepancies that were identified by the NLP algorithm, we found more than 90% of the discrepancies were associated with a “Delete” recommendation. In other words, delabeling plays a major role in eliminating allergy discrepancy. There are several reasons that may explain the accumulation of potentially mislabeled allergies. First, since the challenge test results are most often negative, the allergen will need to be deleted rather than added to the list; selecting “Delete” may be contrary to intuition and require additional steps in the workflow that hinder physicians from deleting allergens (12). Second, physicians tend to be reluctant to remove allergens from the allergy list. During manual review, we found physicians may record a negative challenge test result in the comment field of an allergen instead of removing the allergen directly. Because these allergens remain on the allergy list, the allergen may continue to trigger unnecessary allergy alerts thereafter. In these scenarios, the NLP algorithm can serve to alert physicians to inappropriate allergy discrepancies and prompt them to correct EHR allergy records in a timely manner.

NLP is a powerful tool that has a broad range of uses, including de-identification of sensitive data, early identification of diseases/conditions, detection of health complications, and information extraction from clinical narratives in EHRs (21–24). In our study, we confirmed that the use of NLP algorithms can help clinicians to identify and interpret challenge test results to a greater effect than flowsheet information alone. Only 42% of challenge test results can be determined using flowsheets due to incomplete documentation of allergy data in the EHR. While more information is available in clinical notes, clinical notes, like most unstructured data sources, are not easily manually reviewed, as clinicians will need to spend time on searching the relevant notes in the EHR first and then read through the notes to find the results. The use of NLP allows for efficient analysis of clinical notes without sacrificing clinical resources including time. Moreover, by combining flowsheet information with NLP interpreted results from clinical notes, we can interpret the greatest number of test results (76.0%) and challenge test discrepancies (78.4%). This dramatic improvement (2.74 times greater) compared to the use of flowsheets alone establishes the critical role of NLP in the task of allergy reconciliation for challenge test discrepancies. Moreover, we also want to emphasize the importance of physician's role in the allergy reconciliation process. Our tools do not replace but to assist physicians to perform the task efficiently and accurately.

More broadly, our allergy reconciliation initiative presents a highly promising, practical solution that addresses difficulties related to reconciliation and delabeling allergies in EHR, including shortage of staff, training costs, and adaptation to EHR technology (8, 9).

### Limitation

While we have attempted to apply the NLP algorithm to a larger challenge test notes cohort without filtering by the length of the note, the NLP algorithm cannot reliably process short notes (i.e., <1,000 characters in length). After manually reviewing randomly selected short notes, it was evident that they provided variable, limited information. The majority (>90%) of the reviewed short notes included messages like, “Patient had 2 step challenge to Bactrim. Please review flow sheet for documentation.” Therefore, in most cases, the results were only recorded in the flowsheet. There are also a few short notes that may contain valuable information; however, our NLP algorithm is not adapted to capture information from both short, concise notes and long notes without increasingly identifying false discrepant cases since they have different patterns of sentence structure. Nevertheless, the NLP algorithm identified 71% (10 out of 14) allergy discrepancies in the random sample, which is higher than the percentage of allergy discrepancies the flowsheet included (29%; 4 out of 14).

Additional limitations include several patient scenarios that are difficult to interpret using the NLP algorithm. Since our algorithm relies on the clinical notes for an outpatient encounter, we may miss allergy cases when no allergic symptoms are identified or mentioned during the clinic visit itself. The module is also unable to process equivocal data and thereby provide a reliable recommendation because these cases often require another clinic visit to conclusively confirm the challenge test result. Given the absence of data in certain clinical notes and the aforementioned limitations, the NLP may not identify all allergy discrepancies. However, the dramatic improvement in identifying discrepancies relative to using only flowsheets along with its high precision and recall demonstrates the strength and value in applying NLP for allergy reconciliation and delabeling. Lastly, our system architecture was built based on EPIC EHR system and additional efforts may be needed when applying our tools to other non-EPIC base EHR system. However, the core concept of design, development and implementation would be similar in non-EPIC environment.

## Conclusion

NLP demonstrates the capacity to review patient records and identify allergy discrepancies with drug challenge data in the highly variable real-world environment with unstructured data. By optimizing NLP-based logic to effectively interpret EHR information, the NLP-based tool not only supports clinical decision making but also advances patient quality and safety. Future research must continue to validate the use of NLP for allergy reconciliation on a large-scale to contextualize its benefits and shortcomings in clinical care across various clinical and health care settings.

## Data Availability Statement

The Protected Health Information (PHI) data is not publicly accessible and only use for research within the institution after IRB approval. Requests to access the datasets should be directed to LZ, lzhou@bwh.harvard.edu.

## Ethics Statement

The studies involving human participants were reviewed and approved by Brigham and Women's Hospital. Written informed consent from the participants' legal guardian/next of kin was not required to participate in this study in accordance with the national legislation and the institutional requirements.

## Author Contributions

Y-CL extracted, analyzed the data, built the logic, developed the NLP algorithm and evaluated the performance of the tool. Y-CL and SV performed the chart review and drafted the manuscript. SB assist the NLP logic, concept mapping and data validation. DS generated, validated the mapping tables for the allergen and medication. KB, FG, and DS provided clinical domain knowledge for the recommendation mechanism, user interface and clinical workflow. LZ designed the study, supervised the experiment, critically revised and finalized the manuscript. All authors approved the final version of the manuscript.

## Funding

This research was supported with funding from Agency for HealthCare Research and Quality grant R01HS025375.

## Conflict of Interest

The authors declare that the research was conducted in the absence of any commercial or financial relationships that could be construed as a potential conflict of interest.

## Publisher's Note

All claims expressed in this article are solely those of the authors and do not necessarily represent those of their affiliated organizations, or those of the publisher, the editors and the reviewers. Any product that may be evaluated in this article, or claim that may be made by its manufacturer, is not guaranteed or endorsed by the publisher.

## References

[1] Zhou L Dhopeshwarkar N Blumenthal KG Goss F Topaz M Slight SP. Drug allergies documented in electronic health records of a large healthcare system. Allergy. (2016) 71:1305–13. 10.1111/all.12881 26970431 PMC12841114

[2] Wong A Seger DL Lai KH Goss FR Blumenthal KG Zhou L. Drug Hypersensitivity Reactions Documented in Electronic Health Records within a Large Health System. J Allergy Clin Immunol Pract. (2019) 7:1253–60.e1253. 10.1016/j.jaip.2018.11.023 30513361 PMC6456421

[3] Iammatteo M Alvarez Arango S Ferastraoaru D Akbar N Lee AY Cohen HW. Safety and outcomes of oral graded challenges to amoxicillin without prior skin testing. J Allergy Clin Immunol Pract. (2019) 7:236–43. 10.1016/j.jaip.2018.05.008 29802906

[4] Sacco KA Bates A Brigham TJ Imam JS Burton MC. Clinical outcomes following inpatient penicillin allergy testing: a systematic review and meta-analysis. Allergy. (2017) 72:1288–96. 10.1111/all.13168 28370003

[5] Tucker MH Lomas CM Ramchandar N Waldram JD. Amoxicillin challenge without penicillin skin testing in evaluation of penicillin allergy in a cohort of Marine recruits. J Allergy Clin Immunol Pract. (2017)5: 813–5. 10.1016/j.jaip.2017.01.023 28341170

[6] Lee RU. Penicillin allergy delabeling can decrease antibiotic resistance, reduce costs, and optimize patient outcomes. Fed Pract. (2020) 37:460–5. 10.12788/fp.0040 33132684 PMC7592897

[7] Chiriac AM Banerji A Gruchalla RS Thong BYH Wickner P Mertes PM. Controversies in drug allergy: drug allergy pathways. J Allergy Clin Immunol Pract. (2019) 7:46–60.e44. 10.1016/j.jaip.2018.07.037 30573422 PMC6466632

[8] Staicu ML Vyles D Shenoy ES Stone CA Banks T Alvarez KS. Penicillin Allergy Delabeling: A Multidisciplinary Opportunity. J Allergy Clin Immunol Pract. (2020) 8:2858–68.e2816. 10.1016/j.jaip.2020.04.059 33039010 PMC8019188

[9] Stone CA Jr Trubiano J Coleman DT Rukasin CRF Phillips EJ. The challenge of de-labeling penicillin allergy. Allergy. (2020) 75:273–88. 10.1111/all.13848 31049971 PMC6824919

[10] Blumenthal KG Wickner PG Hurwitz S Pricco N Nee AE Laskowski K. Tackling inpatient penicillin allergies: Assessing tools for antimicrobial stewardship. J Allergy Clin Immunol. (2017) 140:154–61.e156. 10.1016/j.jaci.2017.02.005 28254470 PMC5496780

[11] Sousa-Pinto B Blumenthal KG Macy E Pereira AM Azevedo LF Delgado L. Penicillin allergy testing is cost-saving: an economic evaluation study. Clin Infect Dis. (2021) 72:924–38. 10.1093/cid/ciaa194 32107530 PMC7958749

[12] Blumenthal KG Ryan EE Li Y Lee H Kuhlen JL Shenoy ES. The impact of a reported penicillin allergy on surgical site infection risk. Clin Infect Dis(2018) 66:329–36. 10.1093/cid/cix794 29361015 PMC5850334

[13] Rimawi RH Shah KB Cook PP. Risk of redocumenting penicillin allergy in a cohort of patients with negative penicillin skin tests. J Hosp Med. (2013) 8:615–8. 10.1002/jhm.2083 24106225

[14] Zhou L Plasek JM Mahoney LM Karipineni N Chang F Yan X. Using Medical Text Extraction, Reasoning and Mapping System (MTERMS) to process medication information in outpatient clinical notes. AMIA Annu Symp Proc. (2011) 2011:1639–48. 22195230 PMC3243163

[15] Blumenthal KG Shenoy ES Wolfson AR Berkowitz DN Carballo VA Balekian DS. Addressing Inpatient Beta-Lactam Allergies: A Multihospital Implementation. J Allergy Clin Immunol Pract. (2017) 5: 616–25.e617. 10.1016/j.jaip.2017.02.019 28483315 PMC5484001

[16] Lutfeali S DiLoreto FF Alvarez KS Patel SV Joshi SR Tarver SA. Maintaining penicillin allergy delabeling: A quality improvement initiative. J Allergy Clin Immunol Pract. (2021) 9:2104–6.e2102. 10.1016/j.jaip.2021.01.005 33482418

[17] Shenoy ES Macy E Rowe T Blumenthal KG. Evaluation and management of penicillin allergy: a review. JAMA. (2019) 321:188–99. 10.1001/jama.2018.19283 30644987

[18] Chen JR Tarver SA Alvarez KS Tran T Khan DA. A proactive approach to penicillin allergy testing in hospitalized patients. J Allergy Clin Immunol Pract. (2017) 5:686–93. 27888034 10.1016/j.jaip.2016.09.045

[19] Blumenthal KG Peter JG Trubiano JA Phillips EJ. Antibiotic allergy. Lancet. (2019) 393:183–98. 10.1016/S0140-6736(18)32218-9 30558872 PMC6563335

[20] Blumenthal KG Huebner EM Fu X Li Y Bhattacharya G Levin AS. Risk-based pathway for outpatient penicillin allergy evaluations. J Allergy Clin Immunol Pract. (2019) 7:2411–4.e2411. 10.1016/j.jaip.2019.04.006 30981747 PMC6733651

[21] Stubbs A Filannino M Uzuner O. De-identification of psychiatric intake records: Overview of 2016 CEGS N-GRID shared tasks Track 1. J Biomed Inform. (2017) 75S:S4–S18. 10.1016/j.jbi.2017.06.011 28614702 PMC5705537

[22] Topalovic M Das N Burgel PR Daenen M Derom E Haenebalcke C. Artificial intelligence outperforms pulmonologists in the interpretation of pulmonary function tests. Eur Respir J. (2019) 53:1801660. 10.1183/13993003.01660-2018 30765505

[23] Karhade AV Bongers MER Groot OQ Kazarian ER Cha TD Fogel HA. Natural language processing for automated detection of incidental durotomy. Spine J. (2020) 20:695–700. 10.1016/j.spinee.2019.12.006 31877390

[24] Shung D Tsay C Laine L Chang D Li F Thomas P. Early identification of patients with acute gastrointestinal bleeding using natural language processing and decision rules. J Gastroenterol Hepatol. (2021) 36:1590–7. 10.1111/jgh.15313 33105045 PMC11874507

